# A Digital Atlas of the Dog Brain

**DOI:** 10.1371/journal.pone.0052140

**Published:** 2012-12-20

**Authors:** Ritobrato Datta, Jongho Lee, Jeffrey Duda, Brian B. Avants, Charles H. Vite, Ben Tseng, James C. Gee, Gustavo D. Aguirre, Geoffrey K. Aguirre

**Affiliations:** 1 Department of Neurology, School of Medicine, University of Pennsylvania, Philadelphia, Pennsylvania, United States of America; 2 Department of Radiology, School of Medicine, University of Pennsylvania, Philadelphia, Pennsylvania, United States of America; 3 Section of Neurology, Department of Clinical Studies, School of Veterinary Medicine, University of Pennsylvania, Philadelphia, Pennsylvania, United States of America; 4 Section of Ophthalmology, Department of Clinical Studies, School of Veterinary Medicine, University of Pennsylvania, Philadelphia, Pennsylvania, United States of America; The University of New South Wales, Australia

## Abstract

There is a long history and a growing interest in the canine as a subject of study in neuroscience research and in translational neurology. In the last few years, anatomical and functional magnetic resonance imaging (MRI) studies of awake and anesthetized dogs have been reported. Such efforts can be enhanced by a population atlas of canine brain anatomy to implement group analyses. Here we present a canine brain atlas derived as the diffeomorphic average of a population of fifteen mesaticephalic dogs. The atlas includes: 1) A brain template derived from in-vivo, T1-weighted imaging at 1 mm isotropic resolution at 3 Tesla (with and without the soft tissues of the head); 2) A co-registered, high-resolution (0.33 mm isotropic) template created from imaging of ex-vivo brains at 7 Tesla; 3) A surface representation of the gray matter/white matter boundary of the high-resolution atlas (including labeling of gyral and sulcal features). The properties of the atlas are considered in relation to historical nomenclature and the evolutionary taxonomy of the *Canini* tribe. The atlas is available for download (https://cfn.upenn.edu/aguirre/wiki/public:data_plosone_2012_datta).

## Introduction

The domestic dog has served as an experimental model in neuroscience experiments and translational neurology for several centuries. Some of the earliest evidence for specific localization of brain function derived from experiments on dogs by Gustav Fritsch and Eduard Hitzig, who electrically stimulated small regions of the exposed cortex in awake animals [1], [2]. Using similar techniques, Sir David Ferrier identified multiple cortical areas related to the precise control of movement and translated these findings to map the “eloquent” cortex of patients with tumors undergoing neurosurgical procedures [3]. One of the earliest localizations of visual cortex was in the dog, identified using focal lesions [4], and in the early 20th century, the dog was used as a model of traumatic brain injury from missile wounds [5]. Perhaps the most celebrated use of dogs in neuroscience and psychology was the work of Ivan Pavlov that characterized conditioned reflexes [6], [7].

There has been a recent revival of interest in the canine as a model of ophthalmologic and neurologic disease. The dog has become an important model system for inherited retinal disease [8], and gene therapeutic treatment of these disorders (e.g. [9]–[11]). Dogs suffer from age-related cognitive dysfunction, and the associated neuropathology resembles human Alzheimer’s Disease [12]–[14]. The dog has also become a valuable model of inherited leukodystrophies [15], [16], and potential gene therapeutic treatment of lysosomal enzyme deficiencies [17], [18].

The dog continues to be an essential model of social cognition. Recent behavioral work in canines has examined the extent (and variability) of cognitive skills in different dog breeds, such as tracking cues [19], pointing gestures [20], and even “word learning” [21]. This interest in behavior and sensory function has led to a small but growing number of studies using functional and anatomical magnetic resonance imaging to study the canine brain. A set of early studies showed that visual stimulation in the anesthetized dog could produce measurable changes in blood oxygen level dependent (BOLD) fMRI signal from the canine visual cortex [22], [23]. Subsequently, the recovery of cortical responses following treatment of retinal disease by gene therapy was studied in the dog model [24]. A recent fMRI study has examined the neural correlates of reward mechanisms in the awake dog [25].

As the number of MRI-based studies of the canine brain grows, so does the need for a standard MRI-based template of the dog brain. Such a template allows data from across animals to be registered to a common space to be combined and compared, and facilitates quantitative comparisons of anatomical features. Here we present an atlas of the canine brain that is well suited for this purpose. This atlas is a diffeomorphic [26], population-based average that is composed of a low-resolution brain volume to be used for automated registration and skull-stripping, and a co-registered, high-resolution volume for data display and referencing of effects to the cortical surface.

## Materials and Methods

### Animals

A total of 15 dogs with mesocephalic (mesaticephalic) conformation were studied; all were purpose-bred, mixed-breed dogs that originated from several breeds having various forms of inherited retinal degenerations [27]. Thirteen of these animals were homozygous *RPE65*-mutants, resulting in severe retinal photoreceptor dysfunction present at birth, later treated successfully with subretinal injections of an adeno-associated viral vector carrying wild-type *RPE65* [24]; the remaining 2 animals were non-affected carriers of the mutation with normal retinal function. Each animal was studied to obtain MRI images of brain anatomy either for the low-resolution, “in-vivo” atlas, or for the high-resolution, “ex-vivo” atlas.

#### Ethics Statement

This study was carried out in strict accordance with the recommendations in the Guide for the Care and Use of Laboratory Animals of the National Institutes of Health. The protocol was approved by the Institutional Animal Care and Use Committee of the University of Pennsylvania (IACUC Protocol #s 803269 and 801870). All procedures were carried out under anesthesia and all efforts were made to minimize discomfort.

### In-vivo Low Resolution Template

#### Magnetic resonance imaging

**Figure 1:**
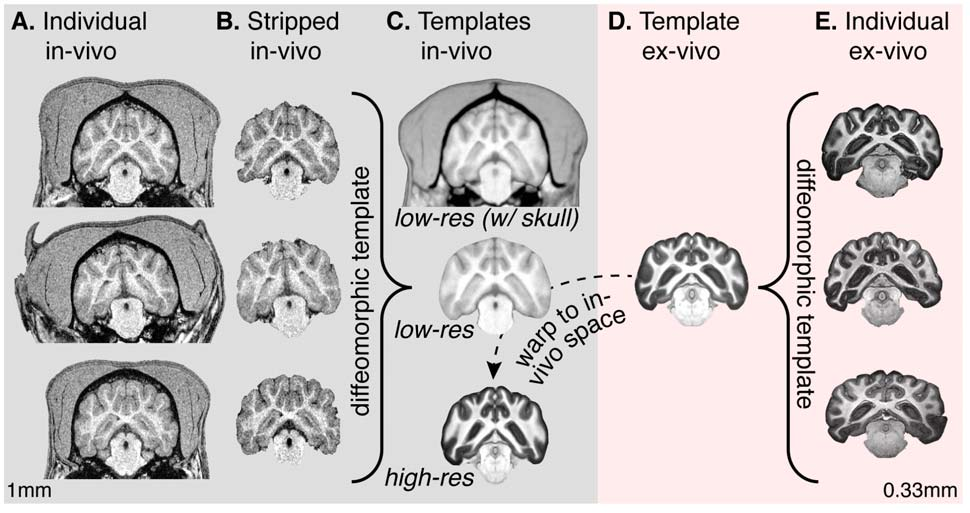
In-vivo low resolution and ex-vivo high resolution templates. (A) In-vivo T1-weighted images from three individual canines, obtained with 1 mm isotropic resolution. (B) Individual in-vivo brains following manual brain extraction. (C) Templates in the in-vivo space. Top is the diffeomorphic average, low-resolution brain including the soft-tissues of the head. Middle is the average, low-resolution, skull-stripped brain. Bottom is the high-resolution, ex-vivo diffeomorphic average following warping to the in-vivo space. (D) The high-resolution, diffeomorphic average of the ex-vivo brains, in the ex-vivo space. (E) Examples of high-resolution, ex-vivo brains scanned at 7 Tesla with 0.33 mm isotropic resolution.

T1-weighted images from seven dogs were used in the creation of the in-vivo template. Two, 15-minute MPRAGE images (1 mm isotropic) were acquired for each animal on a 3 Tesla Siemens Trio (Erlangen, Germany) using a transmit–receive, quadrature volume head coil (USA instruments, Aurora, Ohio). Each dog was anesthetized, and, during image acquisition, also paralyzed and ventilated (for details of anesthesia protocol, see [24]). The two MPRAGE images were subjected to 6-parameter realignment with least-squares minimization and then averaged (Figure 1A).

#### Creation of low-resolution, in-vivo anatomical template

The anatomical image for each animal was segmented to separate the brain from the skull (Figure 1B) using semi-automated, open-source methods available in ITK SNAP (http://www.itksnap.org/pmwiki/pmwiki.php) [28]. A cascade of transformations were then applied to generate an unbiased shape and intensity template using the Advanced Normalization Tools (ANTs) (http://www.picsl.upenn.edu/ANTS/). The initial distribution of the skull-stripped brains was estimated by directly averaging their intensities. The second step involved rigidly registering each image to the intensity distribution using ITK mutual information as a similarity metric. Then a trimmed average of the rigidly registered intensities was used to tighten the distribution [29]. Next, an elastic registration model was applied to find a sharper trimmed average intensity image and a small deformation shape average. The final step used large deformation diffeomorphic image registration and shape averaging to bring all structures of the brains into exact correspondence which yielded the final unbiased intensity average and the final optimal shape anatomy [26]. The middle panel of Figure 1C (labeled *low-res*) is the un-biased, diffeomorphic template average derived from the low-resolution (1 mm isotropic), in-vivo set of brains.

We then created a template in the same space as the *low-res* atlas, which retained the skull and head. To do so, individual skull-striped canine brains were diffeomorphically registered to the *low-res* atlas. The transform generated while warping each individual skull-striped brain to the *low-res* atlas was then used to warp the respective individual MPRAGE image containing the entire head to the *low-res* atlas space. A diffeomorphic image registration and shape averaging was then performed on the set of registered, whole-brain images to create the in-vivo atlas that includes both the brain and soft tissues of the head and skull (Figure 1C; labeled *low-res w/ skull*).

A description of the creation of the *low-res* atlas has been presented previously in abstract form [30].

### Ex-vivo High Resolution Template

#### Brain collection

Brains were collected from a separate group of eight animals following euthanasia (intravenously administered Euthasol; Virbac Animal Health, Ft. Worth, TX) after completion of gene-therapy studies. The skin and muscles overlying the skull were removed, and an oscillating saw used to cut the calvarium which was elevated bluntly with a scalpel handle and removed. The overlying meninges were removed, and then the head was rotated 180° to provide better exposure of the ventral aspect of the brain. All cranial nerves were cut with fine, blunt scissors, and the brain removed with minimal damage. To prevent compression during fixation, the brain was placed in 10% buffered formalin, and 37% formalin stock solution was added until the brain floated just below the fluid surface; paper towels soaked in formalin were placed over the surface to prevent drying from exposure. After 48–72 hours the brains were transferred to 10% formalin solution where they were stored for 2 to 3 months prior to the MRI studies. Prior to MRI scanning, the brains were transferred to phosphate buffered saline (PBS), and the fluid changed every 3–4 days for 3 changes.

#### Magnetic resonance imaging

MRI images were acquired on a 7 Tesla whole body MRI system (Siemens, Erlangen, Germany) with a 32 channel phased-array head coil (Nova Medical, Wilmington, PA). The brain was stored in a cylinder filled with PBS and placed at the bottom of the coil to improve SNR. T1-weighted MPRAGE images were acquired. The brain was covered by two sequentially acquired slabs with the middle area overlapped in both slabs. The resolution was 0.33 mm isotropic, FOV  = 84×84×34.3 mm in each slab, matrix size  = 256×256×104 in each slab, TR  = 3 sec, TI  = 550 ms, TE  = 3.4 ms, flip angle  = 12°, pixel bandwidth  = 370 Hz/pixel, and total scan time  = 12:48 min for each slab. The acquisition was repeated 6 times for signal averaging. While there was no veridical movement of the studied tissues, drift of the image within the field of view can occur with warming of the gradient coils. Therefore, the six MPRAGE images were subjected to 6-parameter realignment with least-squares minimization and then averaged (Figure 1E).

#### Creation of high-resolution, ex-vivo anatomical template

Creation of the *high-res* atlas proceeded in a manner similar to that used for the *low-res* atlas. Each T1-weighted structural MRI of the ex-vivo canine brain was mapped using a cross-correlation registration metric to an optimal template space, defined as the population-specific, unbiased average shape and appearance image derived from a representative population which in this study are the individual ex-vivo brains [29].

Without the confinement of the skull, the ex-vivo brains relax into wider left-right conformation. This is reflected in the diffeomorphic average (Figure 1D). The template ex-vivo brain was therefore mapped using a mutual information registration metric to the in-vivo, *low-res* template. This yielded the final, *high-res* atlas which resides within the in-vivo template space.

#### Canine brain inflated surface

The *high-res* template was then processed using a modified version of the automatic anatomical surface reconstruction pipeline of the FreeSurfer toolkit (http://surfer.nmr.mgh.harvard.edu/) [31], [32]. After automatic tissue segmentation, the images were manually inspected to identify errors in the gray / white matter boundary definition. The gray / white matter intensity differences were very large for the majority of the areas in the canine brain and the corresponding tissue segmentation was generally accurate. However, some regions contained partial volume effects and required manual intervention to demarcate the boundary. Control points for white matter voxels were manually defined in areas of problematic tissue segmentation, and the FreeSurfer pipeline re-invoked to estimate the gray white matter boundary. This was performed iteratively until the segmentation was judged sufficiently accurate. Topological holes in the white matter segmentation produced by the ventricles and hippocampi were manually filled. The two hemispheres of the filled white matter volume were then separated. Separate three-dimensional rendering of each white matter hemispheric volume was created. The surface of the three dimensional rendering is the gray matter / white matter boundary which was then smoothed with a surface smoothing kernel to create a smoothed white matter surface. The white matter surface was then inflated using the standard tools available in FreeSurfer.

## Results and Applications

The complete canine atlas set is composed of co-registered, *low-res* and *high-res* volumetric templates, including a *low-res* template that includes the skull and soft tissues of the head. Further, the *high-res* volumetric template serves as the basis of a cortical surface reconstruction of the canine brain. Below we examine the availability of sub-cortical and cortical detail in the template brain; the pattern, nomenclature, and evolutionary history of canine cortical surface topology; and suggested processing approaches for use of the canine atlas.

### Detail

**Figure 2:**
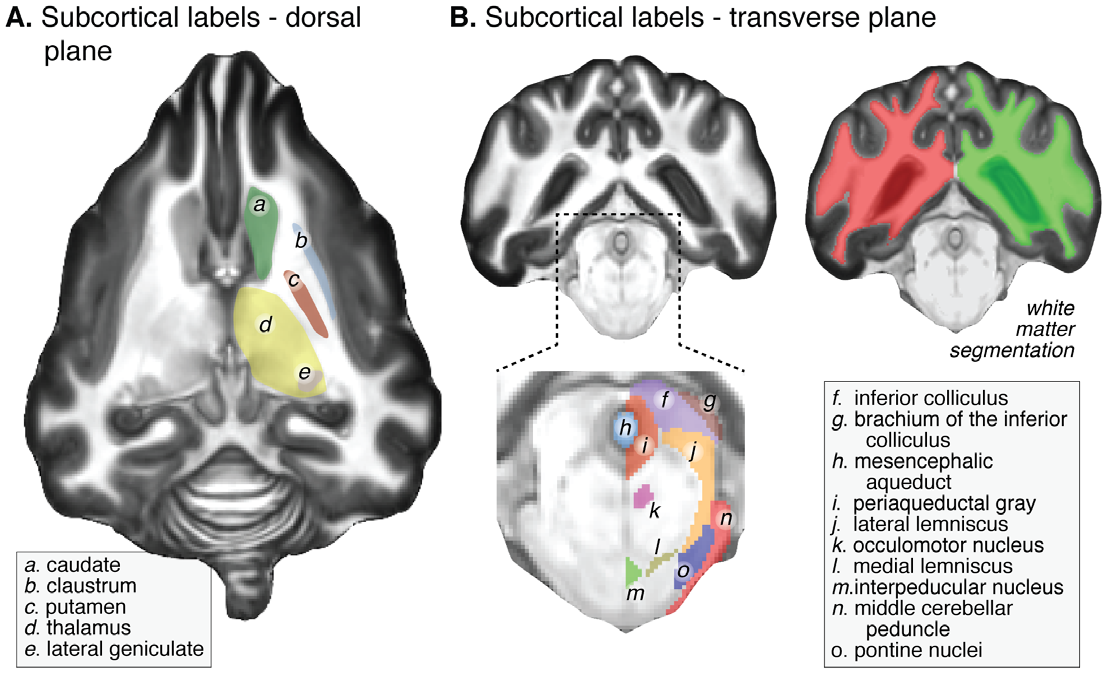
Representative slices of the high resolution ex-vivo template demonstrating labeled cortical and subcortical structures. (A) Dorsal plane (horizontal) slice through the basal ganglia and thalamus. The fine structure of both the lateral geniculate nucleus and head of the hippocampus can be seen in this population average image. (B) Expanded and contrast-enhanced coronal slice through the brainstem, and illustration of white matter tissue segmentation.

In the high resolution (0.33 mm isotropic), ex-vivo MRI images collected with 2½ hours of scanning at 7 Tesla, excellent contrast was available between gray and white matter. While individual differences in anatomy would be expected to induce smoothing of the high-resolution images when averaged across subjects, shape-based diffeomorphic registration matches tissue types prior to averaging. Consequently, the fine detail present in individual, high-resolution brain images is well preserved in the average atlas. Figure 2 illustrates some of the subcortical and brainstem anatomical features that are visible in the atlas. Notable is the clear appearance of the claustrum (Figure 2A, label *b*), which is a thin strip of gray matter located between the external and extreme capsules, and the preservation of the folia of the cerebellum. For some of these structures (e.g., the thalamus seen in axial view in Figure 2A, label *d*) further structure is readily apparent.

In the brainstem, differentiation between gray matter and white matter structures can also be seen. We have labeled some of these anatomical features (Figure 2B, following the [33], [34]). Our goal in doing so is not to provide a comprehensive atlas of all brainstem structures (for an excellent reference for this purpose, see Palazzi [34]) but instead to illustrate that the diffeomorphic average contains sufficient anatomical detail to support such efforts.

The imaging contrast between the gray and white matter enabled the definition of a white matter tissue segmentation (Figure 2B, right). This was iteratively edited to ensure that the white matter volume in each hemisphere was a continuous volume without topological defects, and thus may be expressed as a continuous cortical surface within FreeSurfer.

### Canine Cortical Surface Topology

**Figure 3:**
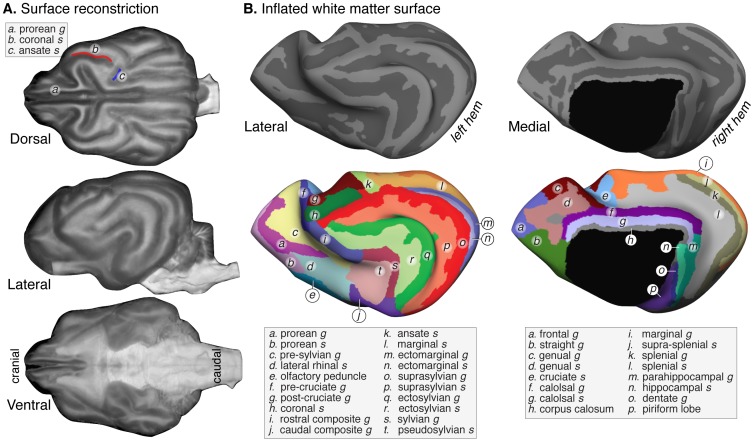
Surfaces and labels. (A) Reconstruction of the canine brain surface from the *high-res* atlas, viewed in three orientations. The location of the prorean gyrus, coronal sulcus (red) and ansate sulcus (blue) is indicated on the dorsal view. (B) Lateral and medial views of the inflated white matter surface with sulci and gyri labeled. The dark gray structures are the sulci and the light gray regions are the gyri. On the labeled surfaces, the sulci are colored in less saturated colors and gyri in saturated colors.

We produced a surface rendering of the canine brain from the *high-res* atlas (Figure 3A). Next, following segmentation of the topologically corrected white matter, a surface based reconstruction of the canine hemisphere was performed within FreeSurfer. The resulting inflated view of the cortical surface (Figure 3B) allows the continuous cortical sheet to be seen, including cortex normally obscured within the sulcal depths. We labeled the sulci and gyri on the cortical surface, generally following the nomenclature of Miller et al [35], [36]. It should be noted that the olfactory bulb, which is a prominent feature in the canine brain, is absent in this ex-vivo atlas as this structure was transected in removal of the brain.

There are disagreements of nomenclature for the canine cortical surface. A prominent variation regards the most medial, dorsal, caudal gyrus. We have adopted the label “marginal gyrus” [37]–[39] instead of the alternate “lateral gyrus” [40]–[44] for this structure, which is the location of primary visual cortex in the dog (by homology to the cat [45] and sheep [46]. Cohn and Papez [47] adopted the term “calcarine sulcus” for the structure that we have labeled the “splenial sulcus” and further described a “posterior calcarine fissue”, extending caudally from the splenial sulcus in approximately half the dogs they studied. We did not observe this structure in our atlas, although we cannot exclude that this is a breed specific difference.

The pattern of cortical folding has been used to provide taxonomic organization of the family Canidae (which includes dogs, wolves, and foxes, among many other extant and extinct species). Based upon the observed size of the prorean gyrus (also termed the proreal gyrus; Figure 3A), Huxley [48], and subsequently Radinsky [49], divided canids into dogs and foxes. Lyras [50], [51] studied endocasts of 29 different living and extinct species of the Canini subfamily (which includes wolves and dogs, but not foxes), and suggested that the overall conformation of the coronal and ansate sulci (Figure 3A) assumes one of four basic patterns which distinguishes among the genera within Canini. The appearance of these sulci in our atlas best corresponds to the “orthogonal” arrangement. This, along with the well formed and elongated prorean gyrus in the atlas, is as expected in the genus Canis, of which the domestic dog subspecies is a member.

### Application Approaches

In practice, if an experimenter has collected T1-weighted anatomical images and functional data, then the workflow described below can be used to warp individual animal data to the template space for group analyses.

First, the individual subject brain with the skull is registered to the *low-res w/ skull* template (Figure 1C, top) using, e.g., the diffeomorphic warping tool available in ANTS. The resulting transformation matrix is then used to project a binary tissue mask (brain vs. not-brain) from *low-res w/ skull* template back to the individual brain space; the individual brain in template space can be extracted from its skull by applying this binary mask. Manual editing of the segmentation mask may be performed at this step if necessary.

Once a satisfactory result is achieved, the extracted brain in the original, individual animal space is registered to the *low-res* template. The resulting transformation matrix may be applied to raw functional data or statistical maps in the original space as well.

We expect the *low-res* template to serve as the best target for registration of anatomical images collected in-vivo in individual animals, as these will be the most similar in contrast and detail. Because the *low-res* and *hi-res* atlases are in register, data may be referred to the *hi-res* atlas following registration to the template space, and further displayed on the cortical surface reconstruction.

## Discussion

The canine atlas was created using diffeomorphic registration of a population of brains, initialized with an intensity average. This approach provides the benefit of shape-based averaging, which guarantees that tissues are in correspondence prior to averaging, while avoiding the bias of using one individual from the set as a template norm [26]. Consequently, the canine digital atlas has two important properties. First, the image appearance is not driven by any specific anatomical structure (as no manual landmarking was required); and second, the image shape is independent of any individual’s anatomical coordinate system [52]. The automated image registration methods used for the generation of the templates assume that the structural correspondences are correct between the images of the different canine brains. This assumption seems well justified as all the animals studied, although mixed-breed, were of a common genetic background [27], and thus would be expected to have similar cortical structure.

The high-resolution anatomical images were obtained from ex-vivo brains. Because the brain changes shape when freed from the confines of the skull, the ex-vivo atlas is an imperfect target for registration of in-vivo images. To mitigate this limitation, we created a volumetric brain template from lower-resolution, in-vivo brain images, and then transformed the ex-vivo average to the in-vivo space. Presuming that the alteration of brain shape produced by skull removal is well captured by the plastic deformation used for co-registration of the *low* and *high-res* atlases, this distortion should be fully corrected in the our atlas.

An edge artifact is present in the *high-res* images acquired at 7 Tesla, consisting of a non-uniform, T1 hyperintense band at the external edges of the gray matter ribbon (best seen in the axial and coronal slices in Figure 2). A possible cause of this band is a long MPRAGE readout that induces different T1 weighting for high spatial frequencies at the gray matter edge. This theory is supported by the finding of two separate bands in the gray matter when a still longer readout was used. An alternative explanation is the effect of chemical fixative [53]. Regardless of the cause, we believe that the hyperintense rim is properly segmented as gray matter, based upon comparison to the low-res images obtained at 3 Tesla. As this artifact is restricted to the outer edge of the cortical sheet, it did not compromise the construction of the white matter label.

We anticipate several applications of the atlas to the analysis of canine neuroimaging data. The atlas may be used to register functional data from different animals to a common anatomical space, allowing group-level inferences (such as was conducted in [24]). Individual differences in brain structure as assessed by different imaging modalities, such as cortical thickness, or diffusion tensor imaging [54], may be related to normal variations of behavior or one of many disease states. Indeed, the canine is a valuable model system for many neurological diseases, including epilepsy [55]; cortical malformations such as lissencephaly [56], [57] and polymicrogyria [58], [59]; dementia [60], [61]; and focal lesions [62], [63]. Given the good registration of high-resolution anatomy with a head model, the atlas can be used to guide source localization of EEG recording in the dog [64].

An important feature of the canine atlas, and a potential limit to these applications, is that it was derived from dogs with mesaticephalic skull conformation, meaning that the skull is “medium” shaped, as opposed to elongated (dolichocephalic; e.g., Greyhounds) or shortened (brachycephalic; e.g., Boxers). Head shape may influence cortical folding in a manner more complex than a simple affine transformation of brain size. Differences in canine skull shape are associated with different sensory and behavioral profiles [65]. For example, the distribution of retinal ganglion cells differs between breeds based on muzzle length [66], presumably related to differences in the extent of binocular vision. Other studies have noted an association between head shape and biomechanical function, with brachycephalic breeds being used as guards and fighters and and dolichocephalic breeds as runners [67]. At the very least, there is evidence that human directed breeding of dogs has produced systematic differences in canine cerebral organization, for example the position of the olfactory lobe [68]. Given breed-specific differences in behavior and skull shape, it is quite possible that cortical topology also differs between breeds and in turn relates to behavioral diversity. Therefore, the canine template offered here should be used with caution in the analysis of data obtained from non-mesaticephalic animals. Conversely, our atlas can serve as a starting point to test for the existence of such differences. For example, brain anatomical images from different breeds may be registered, perhaps using explicit sulcal topology to the surface template, and systematic differences in surface deformation assessed [69].

Another potential limitation of the atlas is that it was derived from animals with a visual impairment. Thirteen of 15 animals used to construct the atlas were born with a severe, congenital form of retinal blindness. This canine analog of Leber’s Congenital Amaurosis is a rod-cone dysfunction caused by mutations in the *RPE65* gene. We might consider that there exists systematically different brain structure in either *RPE65* mutants specifically or animals with congenital blindness generally. We believe the effect upon our atlas is minimal, however. First, RPE65 is not expressed within the central nervous system [70]. Further, the neural retina is not altered in the disease [71], thus preserving the optic nerves and post-chiasmatic anatomy [24]. Second, while congenital blindness (where it has been studied in the human) can alter brain structure, these effects are generally subtle, such as changes in gray matter thickness [72] or the surface area [73] of the striate cortex. In both the current study, and our previous examination of controls and RPE65-mutants [24], no qualitative structural differences were observed.

While the dog brain is gyrencephalic (characterized by the development of sulci and gyri) [74]–as opposed to the lisencephalic (smooth) brains of some mammals and birds–the shape and appearance of the sulci and gyri is generally uniform across individuals [75]; although, see [47]. This implies that diffeomorphic volume based registration approaches, as used here in template construction, are sufficient to register individual brains to the template without the need for surface-based approaches. However, if the degree of variability of cortical folding pattern is subsequently found to be greater between or within breeds, then a surface based registration would be preferred over volume based approaches [76]–[77].

An additional variation of cortical surface topology that remains to be examined is hemispheric asymmetry. Cerebral asymmetry may be a fundamental feature of vertebrates [78], and there is some evidence of anatomical [79], [80] and behavioral laterality [81], [82] in the dog. For example, the right hemisphere is larger than the left hemisphere [79], although specific features, such as the ectosylvian gyrus, is larger on the left [80]. The canine brain atlas we have presented here can serve as the basis of quantitative hemispheric comparisons, using volumetric techniques following mirror reversal [83], [84], or surface-based approaches following the creation of a pseudo-hemisphere that has surface topology intermediate between the left and right [85].

Digital atlases of the healthy (http://vanat.cvm.umn.edu/mriBrainAtlas/) and diseased [39], [86] canine brain have been offered previously. A notable previous effort is the work of Tapp and colleagues [87], which constructed a dog brain template from the average of 192 animals and then used the template in a voxel-based morphometry study of the effect of aging upon the canine brain. The atlas we have created is an advance on these previous efforts in several respects. In addition to basic improvements in voxel resolution and imaging contrast, our atlas has the important property of representing the diffeomorphic central tendency of a group of animals, having both low and high-resolution versions to support an image analysis pipeline, and a surface-based implementation. The ex-vivo images at 7 Tesla provided good spatial and contrast resolution for the identification of anatomical features, which can be difficult to obtain in-vivo using clinical scanners operating at lower field strengths (although see [88], [89] for progress in obtaining in-vivo canine measures at high field). Our atlas is free to use (with appropriate attribution) for academic or commercial purposes, although the atlas may not be distributed for commercial gain. It may be downloaded from our website (https://cfn.upenn.edu/aguirre/wiki/public:data_plosone_2012_datta).
