# Correction: A Digital Atlas of the Dog Brain

**DOI:** 10.1371/annotation/3cb115c6-d2bb-4d11-b52f-71d1a3a6852d

**Published:** 2013-12-10

**Authors:** Ritobrato Datta, Jongho Lee, Jeffrey Duda, Brian B. Avants, Charles H. Vite, Ben Tseng, James C. Gee, Gustavo D. Aguirre, Geoffrey K. Aguirre

Additional National Eye Institute/National Institute Health grants to the eighth author were incorrectly omitted from the Funding Statement. The Funding Statement should read: "This work was supported by a Burroughs-Wellcome Career development award to GKA, grants from the Hope for Vision foundation to GDA and GKA, a grant from the Pennsylvania Lion’s Foundation to GKA, National Eye Institute (NEI) / National Institutes of Health (NIH) grants EY020516 (GKA), -06855 (GDA), and -14579 (GDA), Foundation Fighting Blindness (GDA), and the ONCE International Price for R&D in Biomedicine and New Technologies for the Blind (GDA, GKA). GDA also supported by National Eye Institute (NEI) / National Institutes of Health (NIH) grants EY019304 and EY018241. The funders had no role in study design, data collection and analysis, decision to publish, or preparation of the manuscript." 

